# The TsiogkaSpaeth grid for detection of neurological visual field defects: a validation study

**DOI:** 10.1007/s10072-024-07305-1

**Published:** 2024-01-08

**Authors:** Anastasia Tsiogka, Mark L. Moster, Klio I. Chatzistefanou, Efthymios Karmiris, Evangelia Samoli, Ioannis Giachos, Konstantinos Droutsas, Dimitrios Papaconstantinou, George L. Spaeth

**Affiliations:** 1 1st Department of Ophthalmology, National and Kapodistrian University of Athens, School of Medicine, General Hospital of Athens “G. Gennimatas’’, Athens, Greece; 2 Department of Neurology and Ophthalmology, Wills Eye Hospital and Thomas Jefferson University, Philadelphia, PA USA; 3 Ophthalmology Department, Hellenic Air Force General Hospital, Athens, Greece; 4 Department of Hygiene, Epidemiology and Medical Statistics, Medical School, National and Kapodistrian University of Athens, Athens, Greece; 5 Glaucoma Service, Wills Eye Hospital, Sidney Kimmel College of Medicine, Thomas Jefferson University, Philadelphia, PA USA

**Keywords:** The TsiogkaSpaeth grid test, Visual field, Hemianopia, Neurological disease

## Abstract

**Background:**

The TsiogkaSpaeth (TS) grid is a new, low-cost, and easy to access portable test for visual field (VF) screening which could be used by clinicians in everyday clinical practice. Our study aimed to determine the validity of an innovative screening grid test for identifying neurological disease-associated VF defects.

**Methods:**

We enrolled two groups of participants: We assessed the one eye of ten consecutive adult patients with different types of neurological disease associated VF defects and ten eyes of controls in each group. The TS grid test was performed in each group. Sensitivity, specificity, and positive and negative predictive values of the TS grid scotoma area were assessed using the 24–2 VF Humphrey field analyzer (HFA) as the reference standard.

**Results:**

Sensitivity and specificity of the TS grid test were 100% and 90.91%, respectively. The area under curve was 0.9545 with 95% CI 0.87–1.00. There was a significant correlation between the number of missed locations on the TS grid test and the visual field index of the HFA 24–2 (*r* = 0.9436, *P* < .0001).

**Conclusion:**

The sensitivity and specificity of the TS grid test were high in detecting VF defects in neurological disease. The TS grid test appears to be a reliable, low-cost, and easily accessed alternative to traditional VF tests in diagnosing typical neurological patterns of visual field defects. It would be useful in screening subjects for neurologically derived ocular morbidity in everyday clinical practice and in remote areas deprived of specialized health care services.

## Introduction

Visual field defects are a common manifestation of ophthalmic and neurologic pathological conditions, and perimetry testing is used to document the underlying pattern of field defects they may cause. The central 30 degrees of the visual field covers about 60% of all retinal nerve fibers [1]. Consequently, evaluation of the central visual field tends to reveal the majority of people with visual field loss. In neuro-ophthalmology, apart from functional assessment, perimetry is used in addition, as a diagnostic tool for localizing the site of the lesion and for monitoring the resolution or recurrence of the disease [2]. Hence, diagnostic accuracy of visual field testing is important in neurology for finding any condition affecting the visual pathway so that the diagnosis of neurological pathology with potentially life-threatening consequences is not delayed [3]. Visual fields can be examined with many different techniques, including confrontation visual field testing, tangent screen, Goldmann kinetic perimetry, and automated static perimetry [2].

Current perimeters are accurate, but they have some disadvantages. The examination is a time-consuming process, the devices are bulky, heavy, and expensive, and most of them need specific technology [4]. For visual field testing and analysis, many new portable developments continue to emerge. These promise for home-based monitoring. Despite portability, they are expensive and not widely available [5].

To overcome these problems, we designed a new grid test that has minimal cost and can be used almost anywhere. The TsiogkaSpaeth (TS) grid was designed specifically to analyze VF defects in the central and midperiphery degrees (33.6° height and 43.2° width) surrounding fixation. Our goal is to provide clinicians with a low-cost, easy-to-access, portable test that has a short testing and processing time. The aim of the present study is to determine the validity of the TS grid test for identifying visual field defects in patients with neurological conditions.

## Materials and methods

This study took place in the First University Ophthalmology Department of General Hospital of Athens “Georgios Gennimatas.’’ Patients were selected and informed about the nature of the study and agreed in writing with full awareness of the procedure. The hospital ethics committee approved the survey, in line with the principles of the Helsinki Declaration.

### The TS grid

**Fig. 1:**
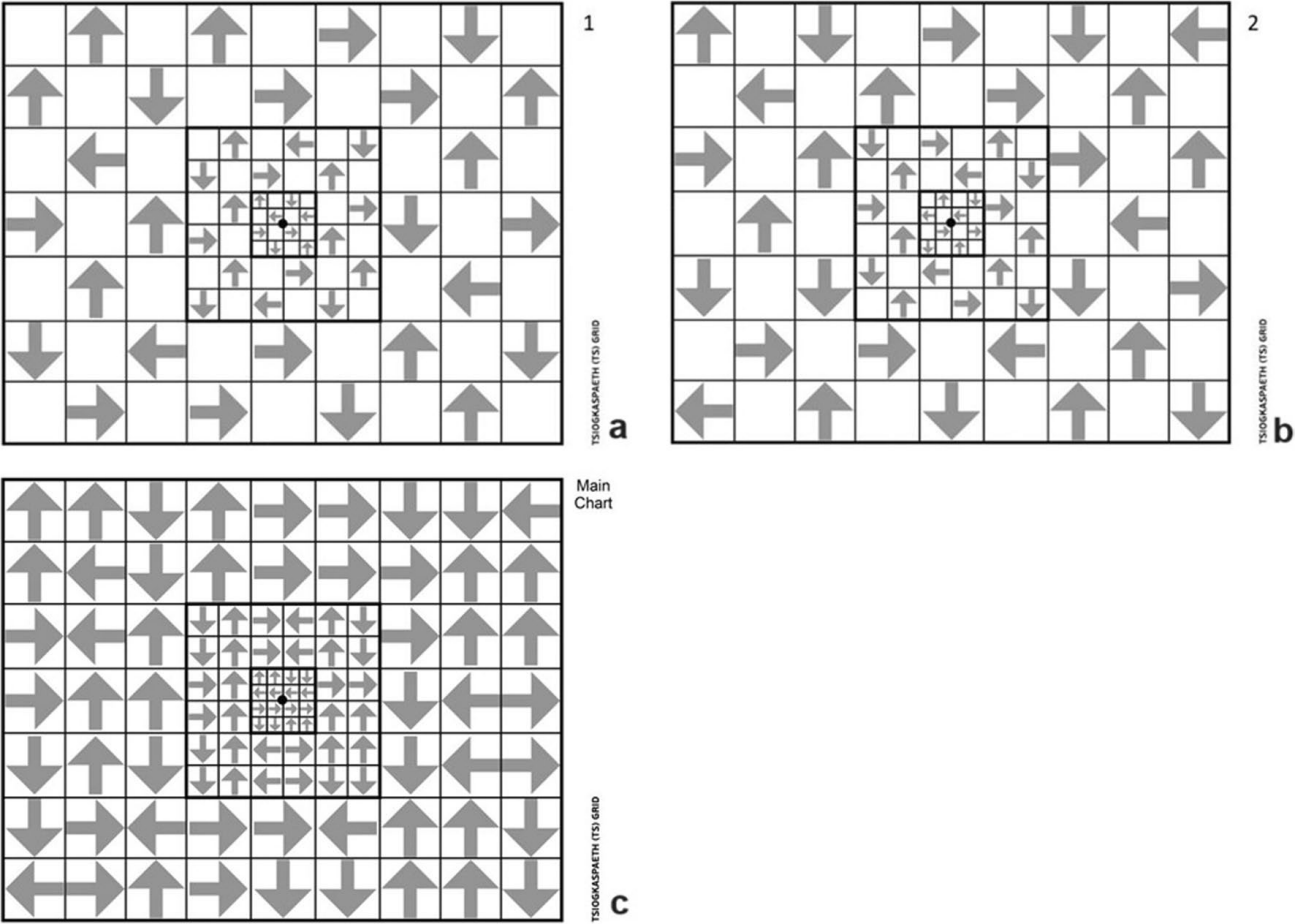
TS grid 1 (**a**), TS grid 2 (**b**), and TS grid main reference chart (**c**) for the clinical examination

The TS grid is a novel optotype, designed to recognize defects in the central and mid peripheral visual field. It is designed to be used on a printed form of a typical A4 paper format presented in horizontal orientation (22.5 cm width and 17.8 cm height). It consists of 54 rectangles of a side length of 2.4 cm, 32 rectangles of side length of 1.2 cm, and 16 rectangles of side length of 0.6 cm in order to exactly fit in the printable A4 form. The smaller rectangles are located in the center of the optotype, surrounded by the medium-sized rectangles, which in turn are surrounded by the larger ones. The concept of gradually increasing the size of the rectangles was employed in order to compensate for the reduced sensitivity in detecting peripheral field defects. In this way, a table is formed, in the shape of a rectangle. These rectangles alternately contain arrows pointing in horizontal or vertical directions separated by empty rectangles in order to facilitate the patient and communication with the examiner. These arrows are pointing in different directions. Recognition of the direction in which each arrow is pointing does not require literacy in any language, does not require recognition of an object, and has only one right answer. It is, also, easily incorporated into a forced-choice paradigm. In addition, the direction of each arrow does not follow a pattern in order to avoid memorization. A small black circle is at the center of the optotype in order to be utilized as the fixation point. Based on the Amsler grid configuration and design [6], each 5-mm rectangle on the TS grid subtends a visual angle of 1 degree when the chart is held at 30 cm. Therefore, the central area of the TS grid subtends 4.8 degrees horizontally and vertically, the paracentral area 15.4 degrees horizontally and vertically, and the entire TS grid 43.2 degrees horizontally and 33.6 degrees vertically. The TS grid is printed in two different forms. One has the arrows occupying one-half of the sites, where the other has the arrows in the other half on the test’s sites. Each eye is examined in two TS grid tests: the first TS grid is complementary to the second grid as shown in Fig. 1a and b. After having the patient wear their best glasses for viewing at near and leaning on a desk, each participant is asked to view the TS grid monocularly at a distance of 30 cm. The fellow eye is occluded with an eye patch. The examinee is instructed to fixate on the central black spot and to try to perceive the direction of the arrows with his/her peripheral vision. If the patient turns his/her eyes to the arrow, he/she is advised to return to the central area and to try to perceive if he/she finally can recognize it or not. During the first examination, the arrows are presented by the examiner to the patient so that the patient learns to recognize them. Patients are instructed to fixate at the central point of the grid at all times and asked to answer if she/he can see the direction of each arrow that is pointed to by the examiner, starting from the central area to the periphery. It is necessary for the person administering the test to return to each tested area once, in order to verify the first answer. Correct and incorrect responses are recorded by circling (indicating a correct response) or deleting (indicating a false response) on the main reference chart (Fig. 1c) held by the examiner. If the patient cannot see an arrow, the procedure is repeated, and if she/he is not still able to see the arrow, the arrow is deleted from the chart. Ideally, testing with the TS grid test should be done at the beginning of the examination in order to avoid potential fatigue that could affect performance. A total TS score is summated from correctly answered rectangles, with 102 the perfect summed score from all rectangles.

### Study population

This cross-sectional study was conducted in accordance with the Declaration of Helsinki and legal regulations. The study protocol was approved by General Hospital of Athens “Georgios Gennimatas’’ review board and the local ethics committee. Recruiting and assessment took place between January 2023 and May 2023. Written informed consent was obtained from all patients before enrollment and individual information of patients was kept confidential.

Two groups of individuals over 18 years of age were involved in the study. The first group included ten patients with neurological disease-associated VF defects, and the second group included ten healthy subjects with no evidence of neurological pathology and a normal visual field test on 24–2 HVF. Despite the fact that both eyes were tested, we randomly selected which eye would be included in the study utilizing a lottery method. Exclusion criteria were identical for the two groups and comprised of existing diagnosis of other systemic or ocular diseases such as glaucoma, corneal pathology and significant cataract, macular diseases, history of retinal detachment, and best corrected visual acuity (BCVA) less than 20/200.

Demographic information (age, gender) and relevant clinical information were recorded for each patient, including Best Corrected Visual Acuity (BCVA), slit-lamp biomicroscopy, and stereoscopic optic nerve head examination using + 78D Volk lens and 24–2 visual fields with Humphrey field analyzer (HFA II), from Carl Zeiss Meditec (Dublin, CA, USA) was performed and recorded. All evaluations were done in a masked fashion by two independent examiners. The Humphrey visual field examination and the TS grid examination were performed the same day by two different examiners in order to avoid biases. The two field tests were evaluated by two experienced clinicians in order to confirm agreement. Patients performed the TS grid and VF tests before any eye examination, because applanation tonometry and pupillary dilation could affect their performance. We used the same examination room with the same lighting conditions (400 lx) for performing the TS grid test in all patients. Two TS grid tests are required for the complete examination, one complementary to the other as outlined above.

### Statistical analysis

The normal distribution of demographic and clinical information was assessed by plots and corresponding statistical tests (Kolmogorov–Smirnov/Shapiro–Wilk test). Normally distributed continuous values were summarized by mean and standard deviation (SD) and discrete data by number (*N*) and percentage (%). Sensitivity, specificity, positive predictive value, and negative predictive values of the TS grid tests were calculated by using the 24–2 HVF test as a standard. Receiver operating characteristic (ROC) analyses were performed with the calculated area under the curve (AUC) as overall measure of fit. To determine the relationship between TS grid score and 24–2 VF results, linear regression analysis was performed between TS grid score and VFI parameter of 24–2 VF HFA. Statistical significance was set at *P* < 0.05. Multivariate linear regression analysis was performed to determine the relationship between TS grid and VFI parameter of 24–2 VF HFA adjusting for other confounders accounted for age or sex separately because of the small sample size. Analysis was conducted in the Stata statistical software package version 13 (STATA Corp., College Station, TX).

## Results

A total of 20 patients were enrolled. One eye of each patient was studied. The mean ± standard deviation (SD) of age was 41.85 ± 16 (range 20–78) years and 50% were males. There were two patients with superior homonymous hemianopia, three with homonymous right hemianopia, two with homonymous left hemianopia, two with homonymous right superior quadranopia, one with homonymous right inferior quadranopia, and ten controls.

**Fig. 2:**
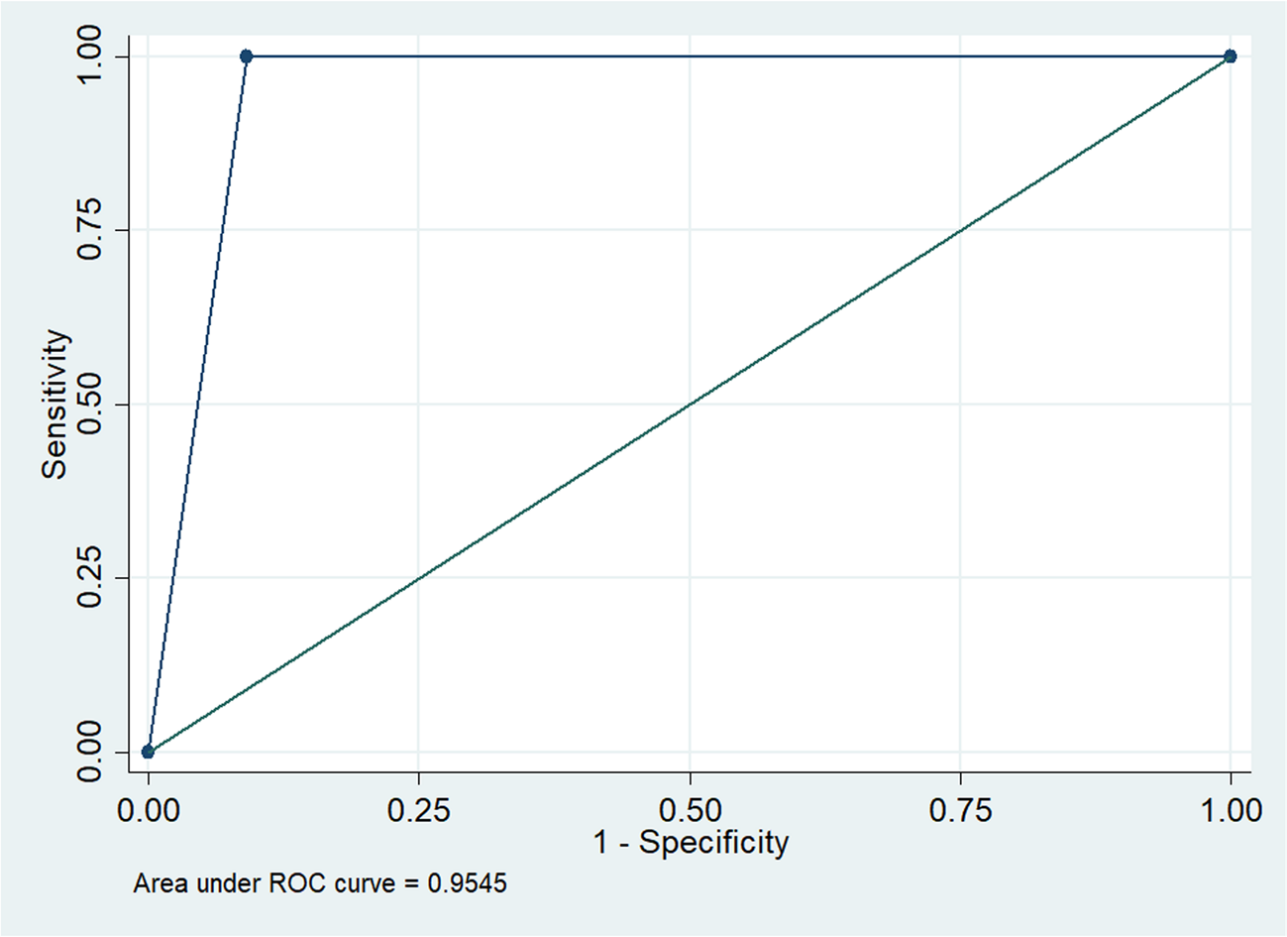
TS grid ROC curve (blue line) and HVA 24–2 ROC curve (green line) for detection of neurological disease-associated VF defects

Among ten eyes with normal 24–2 VF test, nine had a normal TS grid test and one had an abnormal TS grid test. Among 10 eyes with abnormal 24–2 VF test, all ten had an abnormal TS grid (Table 1), with the pattern of the defect being identical between the two evaluation methods. Sensitivity and specificity of the TS grid test defect pattern were 100% and 90.91%, respectively. The AUC for TS grid examination was 0.9545 with 95% CI 0.87–1.00 (Fig. 2).

**Table 1:** 24–2 Humphrey SITA standard visual field test and TS grid test results

		TS grid		
		Abnormal	Normal	Total
HVF 24–2	Abnormal	10	0	10
	Normal	1	9	10
	Total	11	9	20

**Fig. 3:**
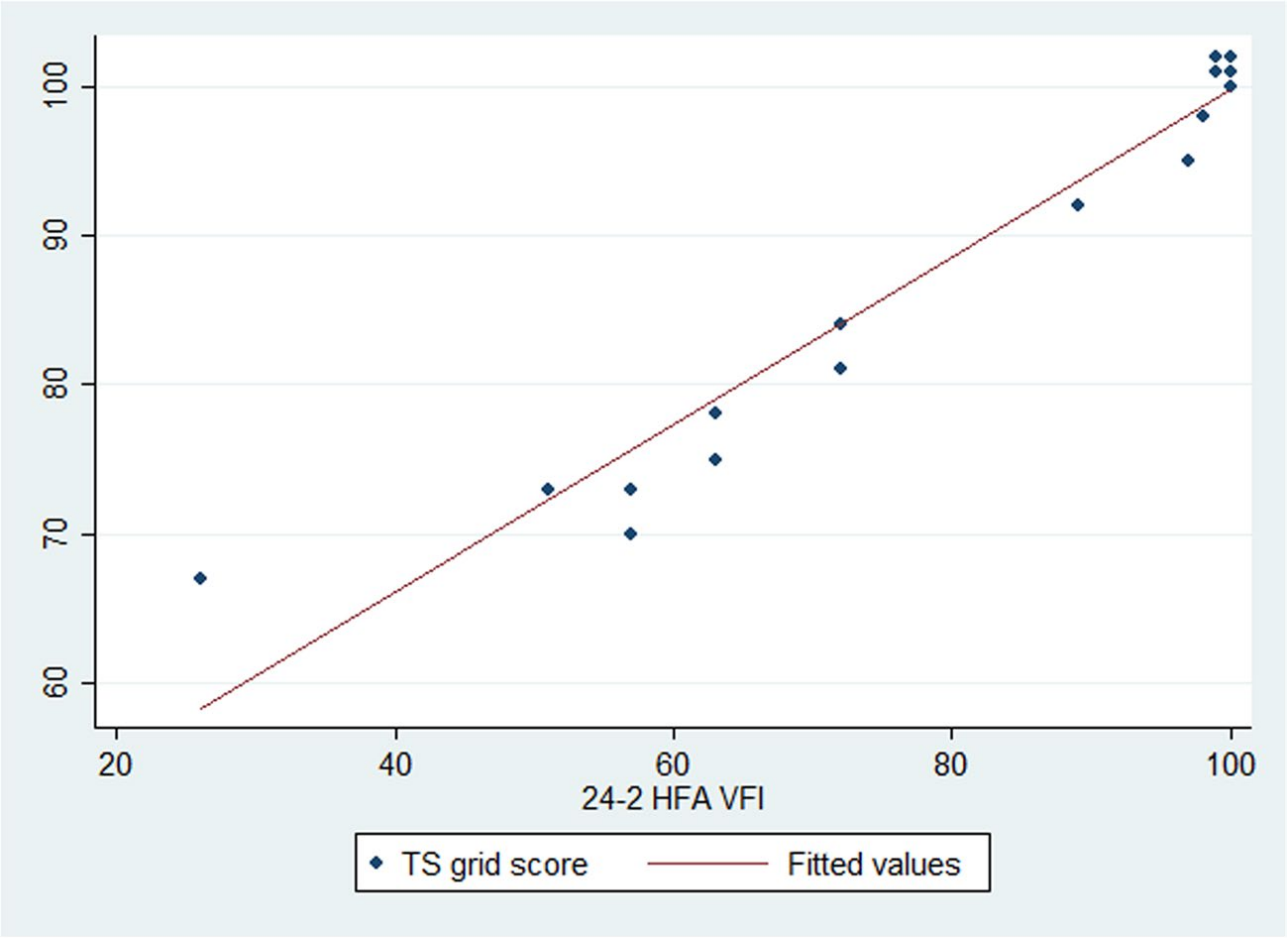
Scatter plots of the TS grid score and HFA 24–2 visual field parameters

**Fig. 4:**
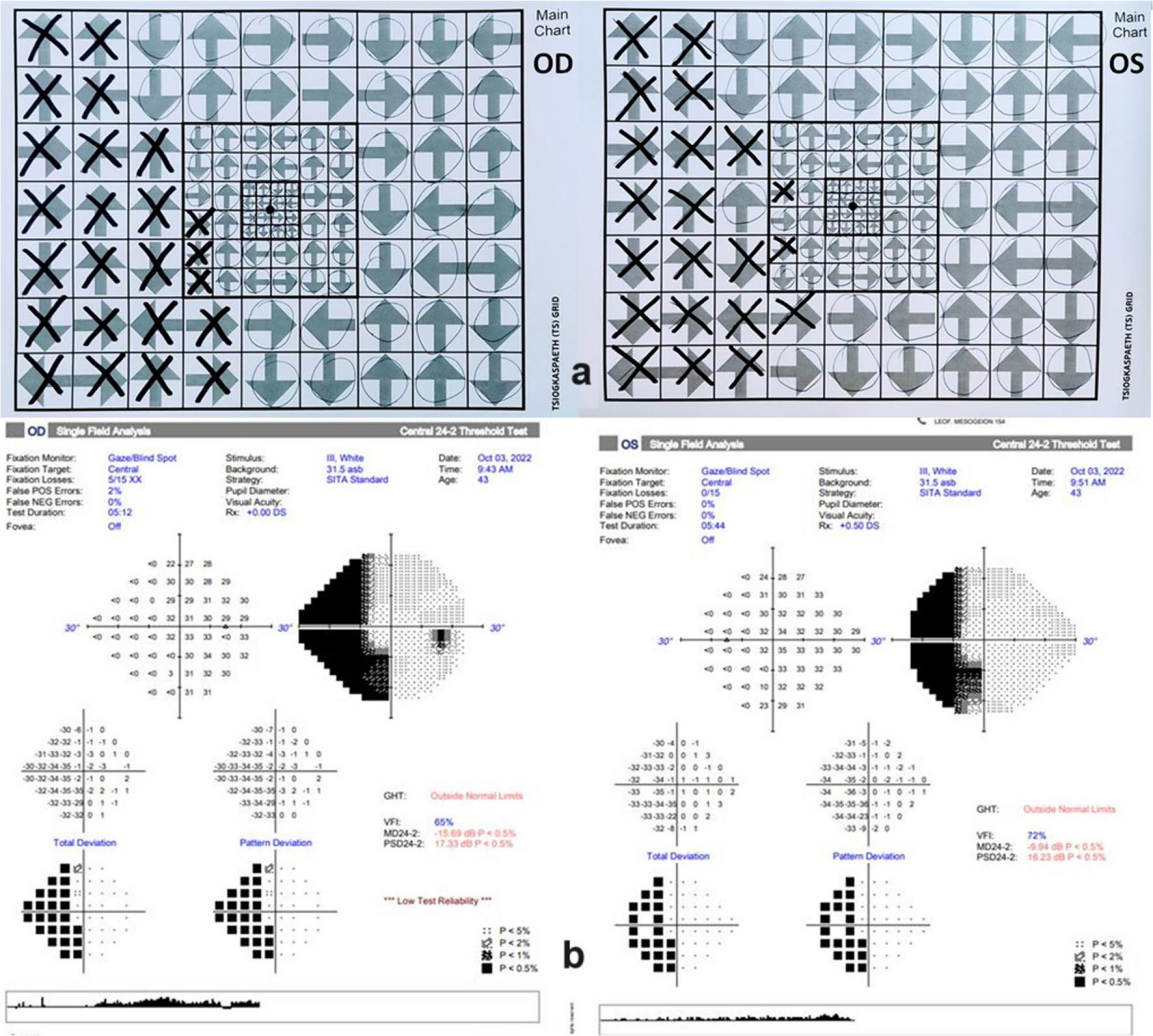
The TS grid presentation pattern (**a**) and test results in comparison to the HFA 24–2 test (**b**) of a patient with left homonymous hemianopia after an ischemic stroke at occipital lobe. For demonstration purposes, both eyes of this subject are included in this image; however, only left was selected for the analysis

There was a significant correlation between the number of the correctly answered rectangles in the right location on the TS grid test and of the HFA 24–2 VFI of 24–2 (*r* = 0.9436, *P* < 0.0001). Results are demonstrated in Fig. 3. The TS grid examination took an average of 5 min to complete, compared with 7 min that were needed on average per 24–2 Humphrey VF test. Figure 4 shows one example of the TS grid presentation pattern (a) and test results in comparison to the HFA 24–2 test (b) in a patient with right hemianopia. This demonstrates good correlation between the TS test and the results obtained with the 24–2 program of the HFA.

We further applied multivariate linear regression analysis to determine the relationship between the TS grid and VFI parameter of 24–2 VF HFA adjusting for other confounders where there was no change to *r*-squared when we accounted for age or sex separately. The correlation between the number of the correctly answered rectangles in the right location obtained with the TS grid test and with the HFA 24–2 VFI of 24–2 remained significant.

## Discussion

Visual field assessment is important in the initial evaluation and follow-up of pathologies involving the visual pathways [2]. Various techniques can be used for this purpose [2].

The standard confrontation visual field testing technique does not require complicated equipment and can be performed anywhere. The examiner must have normal visual fields [7]. A limitation of confrontation visual field testing is the lack of sensitivity in the identification of specific patterns of field loss [8, 9]. The most widely used method to assess visual field deficits is automated perimetry [10, 11], which has become a mainstay for the assessment of peripheral visual field loss related to various ocular and neurologic disorders. Standard automated perimetry (SAP) has been shown to be adequate in the evaluation of VF defects in patients with underlying neuro-ophthalmic pathology and is now the method of choice for most physicians [2]. However, the technique has some disadvantages; it is a time-consuming process often tiring and demands considerable concentration throughout the test. Moreover, the equipment is neither portable nor available for home use and requires trained personnel. These characteristics limit their use in developing countries, as well as by patients with limitation of mobility [4].

Recent technological advances are giving rise to novel tests of visual function. Online visual field testing is a low-cost method that is promising for screening patient populations with limited access to health care, but there is a need for the use of a personal computer. Another limitation of this test is the difficulty that older people may have in using computers [12].

There are many portable devices designed for objective assessment of visual field loss. The nGoggle is a portable brain-based device for assessment of electrical brain responses associated with visual field stimulation. In a clinic-based setting, it was able to discriminate eyes with glaucomatous neuropathy from healthy eyes and showed adequate test–retest repeatability, suggesting that the device may be useful for longitudinal monitoring patients with neural loss [13]. The Virtual Eye perimeter is a head-mounted, eye-tracking perimeter that is similar to the Humphrey full threshold 24–2 visual field and is operated through a portable Windows computer (laptop or desktop) [14]. The Kasha visual field is a portable automated perimeter which utilizes a virtual reality headset. Early trials comparing this head-mounted perimetry device with the Humphrey field analyzer have found comparable results in terms of field classification [15]. The main disadvantage with these devices is that they are usually expensive and high technology. Their proper use requires training of the operators so that they cannot easily be used in places where highly trained technicians are not available.

The TS grid was designed to overcome the disadvantages of techniques currently used in the primary care setting and to provide health professionals an easy way to screen patients in their everyday practice. The results of the TS grid correlated well with Humphrey field analyzer (HFA II). The sensitivity and specificity of the TS grid test were found to be 100% and 90.91%, respectively, for detecting visual field loss patterns in patients with neurologic pathology. Furthermore, it was found to be statistically positively correlated to the visual field index which is a global age-adjusted index developed by Bengtsson and Heijl in 2008. It is expressed in percentage, where 100% represents a normal visual field and 0% represents a perimetrically blind field [16].

The advantages of our proposed test are that it is easy, simple, and quick and does not depend on any special equipment. It does not require specialized medical personnel as examiners. It takes 4–5 min per eye and can be repeated as many times as needed. Our study showed that the TS grid is an effective tool for assessing visual fields defects in patients with neurological conditions that may produce visual field defects. The TS grid test may be used for screening, detecting or monitoring visual field defects of neurologic origin. Standard automated perimetry may then be used to quantify any defects.

The TS grid is inexpensive, safe, and simple to perform. It can be used in the primary care setting for screening and monitoring patients with visual field defects caused by neurological disease. It could also be used in patients with limited access to health care, in countries where incomes are low, and with patients whose mobility is limited. It may be administered almost anywhere. It may potentially be useful for self-testing.

The main limitation of our study is the small number of patients. A larger sample size of patients is required to reach any general conclusion about the validity of this method. Another limitation is the difficulty of ensuring that the patient maintains fixation on the central fixation point of the grid. Additionally, the grid’s design respects the horizontal and vertical meridians only in the central 15 degrees of the field. The strengths of this study are the close agreement between the results of HVF tests and TS grid tests. This is the initial report of a new method of testing visual fields that does not require high technology.

## Conclusions

The TS grid provides an inexpensive, accurate method of identifying patients with visual field defects caused by neurologic conditions. It can be administered almost anywhere, such as in a doctor’s office, at home, or in the field. While the initial results are highly encouraging, further studies are needed, involving more patients and patients with various stages of disease.

The submitted work is original and has not been considered for submission to other journals.

## Data Availability

Anonymized data are available on reasonable request to the corresponding author.

## Abbreviations

CI: Confidence interval
SITA: Swedish interactive threshold algorithm
HVF: Humphrey visual fields
VF: Visual fields
HFA: Humphrey field analyzer
The TS grid: The TsiogkaSpaeth grid
AUC: Area under curve
VFI: Visual field index
BCVA: Best corrected visual acuity
ROC: Receiver operating characteristics
SD: Standard deviation
